# [^18^F]PBR146 and [^18^F]DPA-714 *in vivo* Imaging of Neuroinflammation in Chronic Hepatic Encephalopathy Rats

**DOI:** 10.3389/fnins.2021.678144

**Published:** 2021-08-16

**Authors:** Xiang Kong, Song Luo, Yun Fei Wang, Gui Fen Yang, Guang Ming Lu, Long Jiang Zhang

**Affiliations:** ^1^Department of Diagnostic Radiology, Jinling Hospital, Medical School of Nanjing University, Nanjing, China; ^2^Department of Nuclear Medicine, Jinling Hospital, Medical School of Nanjing University, Nanjing, China

**Keywords:** hepatic encephalopathy, positron emission tomography, neuroinflammation, translocator protein, cognition

## Abstract

Neuroinflammation is an important pathogenesis of hepatic encephalopathy (HE). The upregulation of translocator protein (TSPO) during neuroinflammation provides an imaging molecular target to evaluate the severity of neuroinflammation in chronic HE rats. [18F]DPA-714 and [18F]PBR146 targeting TSPO are often used for neuroinflammation imaging. This study performed bile duct ligation (BDL) in rats to simulate chronic HE model, tested the behavioral experiments, and conducted [18F]PBR146 and [18F]DPA-714 micro-PET/CT scans followed analyzing the average %ID/g values of the whole brain, brain regions and main organs of subjects. After sacrifice the rats, the blood plasma samples were taken for blood biochemical indexes and plasma inflammatory factor levels examination, the liver and brain specimens were obtained for pathological analysis. The BDL rats showed chronic liver failure with defects in cognition, motor coordination ability and mental state. [18F]PBR146 and [18F]DPA-714 micro-PET/CT imaging results were similar in whole brain of BDL group and Sham group. Besides, some regional brain areas in BDL rats were found abnormal uptakes mainly located in basal ganglia area, auditory cortex, motor cortex, cingulate gyrus, somatosensory cortex, hippocampus, thalamus, midbrain, and medulla oblongata, and these regions also correlated with behavioral alterations. In conclusion, both [18F]PBR146 and [18F]DPA-714 had the similar imaging effects in hepatic encephalopathy models could quantitatively evaluate neuroinflammation load and distribution. The difference brain regions with higher uptake values of radiotracers in BDL rats were correlated with behavioral alterations.

## Introduction

Hepatic encephalopathy (HE) is a serious complication characterized by advanced liver failure and a spectrum of neuropsychiatric impairments ranging from minimal HE to delirium or coma, which are correlated with a poor prognosis (Bajaj, 2018; Kerbert and Jalan, 2020). Systemic inflammation and hyperammonemia attributed to acute and chronic liver failure induce neuroinflammation and brain dysfunction (Butterworth, 2013). Since HE is often accompanied by multiple organ failure and high mortality, this has resulted in little progress in the drug treatment of HE. However, neuroinflammation may offer pathophysiological insight into the underlying mechanisms of cognitive alterations during the development of HE (Bass et al., 2010).

In particular, bile duct ligation (BDL) can induce biliary cirrhosis with hyperammonemia and jaundice after more than 28 days post-surgery, which has provided researchers with an experimental rat model of chronic HE (Butterworth et al., 2009). The translocator protein (TSPO), previously known as peripheral-type benzodiazepine receptor (PBR), was originally identified in peripheral organs and is expressed at low concentrations in healthy brain parenchyma. The upregulation of TSPO occurs when microglia are activated during neuroinflammation, which has 10 min at 50 min to serve as a clinical biomarker for neuroinflammation in neurological diseases (Rupprecht et al., 2009; Setiawan et al., 2015; Jaipuria et al., 2017). Radiolabeled ligands targeting TSPO for positron emission tomography (PET) help visualize the distribution of TSPO *in vivo* in order to detect activated microglia in the brain. Furthermore, they have been considered a novel biomarker for neuroinflammation, and more than 40 TSPO compounds labeled with [^11^C] or [^18^F] have been identified over the past two decades (Alam et al., 2017).

Previously, [^18^F]DPA-714 and [^11^C]PK11195 imaging has been performed in acute and chronic HE rat models. Studies demonstrated that [^18^F]DPA-714 may be a more suitable agent in HE experimental models (Kong et al., 2016; Luo et al., 2018). Another radiotracer [^18^F]PBR146 (N,N-diethyl-2-(2-(4-(3-[^18^F] fluoropropoxy)phenyl)-5,7-dimethylpyrazolo[1,5-a]pyrimidin-3 -yl)acetamide) was proposed in 2007 and was found to have high *in vitro* affinity and selectivity for PBR (Fookes et al., 2008). While [^18^F]PBR146 has not been applied to a HE rat model, we performed [^18^F]PBR146 and [^18^F]DPA-714 micro-PET/CT imaging in a chronic HE rat model with high levels of neuroinflammation. We hope that the [^18^F]PBR146 might have the same imaging effect for chronic HE as [^18^F]DPA-714. We also performed behavioral assessments, biochemical indexes measurements, inflammatory factor level examination, and pathological analysis to determine the relationships among neuroinflammation, cognitive function, and systemic inflammation in BDL rats.

## Materials and Methods

### Animal Model

A total of 20 male (excluding the effects of estrogenic hormones) Sprague-Dawley rats (160–190 g) were used with the permission of the local animal experimental ethics committee. Experimental protocols were conducted in accordance with the guidelines for laboratory animal care and use following the ARRIVE guideline. The rats were freely fed commercial rat food and water sterilized by ^60^Co. Controlled conditions were maintained at 18–22°C, 40–60% relative humidity, and a noise level of < 60 dB at a 12 h (h)/12 h day/night cycle. All subjects were randomized into two groups: sham group (*n* = 8) or BDL group (*n* = 12). The sample size was determined by considering the mortality rate according to previous studies (Kong et al., 2016; Luo et al., 2018). Details regarding the BDL procedure are described in the Supplementary Materials. Sham group rats were handled similarly except for the bile duct ligation and abscission (Rodrigo et al., 2010; Kong et al., 2016).

### Behavioral Studies

The behavioral studies involved the rotarod test, beam walking test, and motor activity experiment performed 2–3 days prior to the micro-PET scans. The tests were performed to evaluate motor coordination, tolerance, locomotor activity, and vertical activity of the rats (Jover et al., 2006; Rodrigo et al., 2010; Agusti et al., 2011; Kong et al., 2016). Detailed procedures are described in the Supplementary Materials.

### Radiosynthesis of Ligands, Micro-PET/CT Scans, and Image Processing

Both [^18^F]PBR146 and [^18^F]DPA-714 can be applied as PET radioligands for TSPO imaging. [^18^F]PBR146 and [^18^F]DPA-714 were synthesized as previously described (Fookes et al., 2008; Lavisse et al., 2015; Kong et al., 2016; Luo et al., 2018). Procedures for the synthesis of [^18^F]DPA-714 and [^18^F]PBR146 are provided in the Supplementary Materials. BDL and sham rats were housed in the same conditions for more than 28 days (Butterworth et al., 2009). Micro-PET/CT scans were implemented using an Inveon small animal scanner (Siemens Preclinical Solution) after completion of the behavior studies.

Rats were fixed in the prone position after anesthetization by isoflurane inhalation (induction: 3%, thereafter: 2–2.5%) in oxygen. Radioactive tracers were injected intravenously into the lateral tail vein. PET data were acquired first by CT data acquisition. PET acquisitions were obtained by scanning for 10 min at 50 min post-radiotracer injection, followed by CT scanning for ∼6–10 min to allow for coregistration of radiotracer uptake with tissues. PET setting parameters were as follows: slice thickness = 0.78 mm, matrix size = 128 × 128, field of view (FOV) = 4 cm × 4 cm, and energy levels of acquisition = ∼350–650 keV (default) (Kong et al., 2016). CT setting parameters were as follows: current = 500 A, voltage = 50 kV, and exposure time = 500 ms (Cochran et al., 2017). Micro-PET/CT scans with [^18^F]PBR146 and [^18^F]DPA-714 were performed for two consecutive days.

Image reconstruction was performed using the Inveon Research Workplace (IRW 3.0, Siemens). PET and CT images were coregistered for correct alignment in three dimensions. Global brain and some organs (e.g., lungs, heart, liver, and kidneys) were drawn on the images for the region of interest (ROI) for quantification. The quantified radioactivity uptake of each ROI was presented as the percent injected dose per gram (%ID/g), calculated by dividing tissue radioactivity with injected dose (assuming the tissue density is 1 g/mL). In addition, regional brain uptake of the radiotracer was evaluated using PMOD software (version 3.7, PMOD Technologies LTD, Zurich, Switzerland). PET images were manually fused with the T2-MRI template after coregistration with CT images. The software then drew 58 ROIs of the brain on the PET images with reference to the MR imaging-based atlas, which yielded corresponding radioactivity concentration values. This processing avoided the effects of peripheral vessels and tissues, which show higher tracer distribution (Cochran et al., 2017; Jiang et al., 2019).

### Biochemical, Histopathological, and Immunohistochemical Characterizations

Rats were anesthetized to collect canthus blood (1–2 mL) into procoagulant tubes in the morning, one day prior to micro-PET/CT scans. Blood samples were centrifugalized at 3,000 × g for 10 min (min) at 4°C, and the supernatant was collected and transferred to the local hospital within 2 h for venous blood ammonia measurement. The day after micro-PET/CT scans, blood samples from the heart cavity (5–10 mL) were collected from each rat after deep anesthetization by 10% chloral hydrate. Determination of liver and renal function indicators were conducted in a clinical laboratory. These biochemical indicators from plasma included total bilirubin, direct bilirubin, indirect bilirubin, total protein, albumin, globulin, alanine aminotransferase (ALT), aspartate aminotransferase (AST), alkaline phosphatase, total cholesterol, high-density lipoprotein cholesterol (HDL-C), low-density lipoprotein cholesterol (LDL-C), urea, creatinine, and uric acid (Nanjing Jiancheng Bioengineering Institute, Jiangsu, China). Finally, 5-hydroxytryptamine (5-HT), interferon-γ (IFN-γ), interleukin 1β (IL-1β), IL-6, IL-10, and tumor necrosis factor alpha (TNF-α) were analyzed from plasma samples using enzyme-linked immune sorbent assay (ELISA) kits (Nanjing Jiancheng Bioengineering Institute, Jiangsu, China).

After blood samples were collected, all rats were transcardially perfused with 100–150 mL of saline, followed by 150 mL of phosphate buffered saline (PBS, *pH* = 7.4) for 20–30 min. The liver, brain, jejunum, ileum, and colon specimens were removed, fixed in 10% buffered formaldehyde, paraffin-embedded, and sliced. The specimens were stained with hematoxylin-eosin (H&E) as previously described (Kong et al., 2016; Luo et al., 2018). In addition, microglia were stained by performing immunohistochemistry of glial fibrillary acidic protein (GFAP, 1:800) and ionized calcium binding adaptor molecule-1 (IBA-1, 1:500) (Wuhan servicebio technology CO.,LTD, Hubei, China) (Dickens et al., 2014; Israel et al., 2016). All histopathological and immunohistochemical slices were scanned using a pathological section scanner. Morphological analysis and cell counts were performed using CaseViewer 1.4 and ImageJ software.

### Statistical Analysis

Data analysis was performed using SPSS 20.0 statistical software (SPSS Inc, Chicago, IL, United States). The normality of continuous variables was confirmed by performing a Shapiro-Wilk test. The normally distributed quantitative variables were expressed as mean ± standard deviation (SD), and the median and interquartile range (IQR) were calculated for non-normally distributed data. Independent-sample *t*-tests and Mann-Whitney tests were performed for sham and BDL group comparisons when appropriate (Kong et al., 2016; Luo et al., 2018). The relationship between behavioral data and radiotracer uptake values in the rat brains was investigated using Spearman’s rank correlation test. *P-*values of <0.05 were deemed statistically significant.

## Results

### Weight, Behavioral Studies, and Biochemical Results

All eight rats in the sham group were alive during the whole experiment, while four rats in the BDL group died due to a biliary fistula or an anesthesia accident. Weights of the sham group rats were significantly higher than the BDL group rats from the first day after operation to the end of the experiment (Supplementary Figure 1). Results of the rotarod test, beam walking test, and motor activity were significantly different between the sham and BDL groups (*P* < 0.05, Table 1), indicating that BDL rats developed significant impairments in coordination, tolerance, and motor activity. Serum ammonia levels in the BDL group (39.43 ± 7.91 μmol/L) were higher than those in the sham group (29.88 ± 4.22 μmol/L, *P* = 0.026). Plasma bilirubin, albumin, globulin, ALT, and AST levels in the BDL group were significantly higher than those in the sham group (*P* < 0.01, Table 1).

**TABLE 1:** Comparisons of behavioral, biochemical, and histopathological measurements between sham and BDL rats.

Parameters	Sham group	BDL group	*P*
**Time on the rotarod (s)**	94.7 ± 21.4	73.5 ± 15.3	0.044*
**Beam walking**			
Cross time (s)	10.9 ± 3.5	22.0 ± 12.1	0.025*
Fault numbers (IQR), n ^§^	1.0 (0, 2.0)	3.5 (1.5, 5.5)	0.010*
**Motor activity**			
Crossovers, n	9.1 ± 5.1	4.1 ± 3.1	0.026*
Rearings (IQR), n ^§^	28.0 (12.0, 40.0)	11.0 (8.3, 15.5)	0.015*
**Biochemical measurements**			
Serum ammonia (μmol/L)	29.88 ± 4.22	39.43 ± 7.91	0.026*
Total bilirubin (μmol/L, IQR) ^§^	2.20 (1.70, 2.50)	111.50 (92.48, 136.95)	0.001**
Direct bilirubin (μmol/L, IQR) ^§^	1.40 (1.00, 1.60)	108.00 (91.45, 131.20)	0.001**
Indirect bilirubin (μmol/L, IQR) ^§^	0.50 (0.30, 1.10)	4.25 (1.25, 5.75)	0.036*
Total protein (g/L)	65.61 ± 3.48	64.68 ± 7.95	0.778
Albumin (g/L)	30.47 ± 2.45	20.93 ± 4.05	< 0.001***
Globulin (g/L)	35.14 ± 1.77	43.75 ± 5.68	0.002**
ALT (U/L)	38.71 ± 6.80	90.00 ± 33.51	0.003**
AST (U/L, IQR)^§^	66.50 (60.50, 74.25)	289.50 (209.00, 462.25)	0.002**
Alkaline phosphatase (U/L)	2.33 ± 0.82	16.38 ± 7.76	0.001**
Total cholesterol (μmol/L, IQR) ^§^	2.00 (1.75, 2.05)	4.92 (3.65, 5.85)	0.001**
HDL-C (μmol/L)	0.84 ± 0.07	0.47 ± 0.23	0.001**
LDL-C (μmol/L, IQR) ^§^	0.18 (0.18, 0.20)	1.61 (1.20, 2.50)	0.001**
Urea (μmol/L, IQR) ^§^	6.10 (5.70, 6.40)	4.95 (3.65, 5.85)	0.032*
Creatinine (μmol/L)	22.63 ± 3.53	24.83 ± 6.52	0.442
Uric acid (μmol/L)	19.43 ± 7.44	42.88 ± 13.86	0.002**
5-HT (pg/mL)	245.11 ± 23.13	481.90 ± 17.06	< 0.001***
IFN-γ (pg/mL)	1049.34 ± 136.37	1980.77 ± 152.95	< 0.001***
IL-1β (pg/mL)	26.82 ± 2.68	42.56 ± 2.22	< 0.001***
IL-6 (pg/mL)	57.13 ± 6.84	135.14 ± 13.94	< 0.001***
IL-10 (pg/mL)	56.48 ± 3.05	107.77 ± 9.07	< 0.001***
TNF-α (pg/mL)	192.48 ± 43.73	326.93 ± 76.68	0.007**
No. of microglia (cells/mm^2^)^†^	29.70 ± 2.68	35.07 ± 1.97	0.006**

Non-normally distributed values are represented as the IQR, and other values are represented as the mean ± standard deviation from at least four rats in each group for the behavioral studies and from at least five rats in each group for the biochemical measurements.

*P < 0.05, **P < 0.01, and ***P < 0.001 were regarded as statistically significant.

*BDL, bile duct ligation; IQR, interquartile range; ALT, alanine aminotransferase; AST, aspartate aminotransferase; HDL-C, high-density lipoprotein cholesterol; LDL-C, low-density lipoprotein cholesterol; 5-HT, 5-hydroxytryptamine; IFN-γ, interferon-γ; IL-1β, interleukin 1β; IL-6, interleukin 6; IL-10, interleukin 10; TNF-α, tumor necrosis factor alpha.*

*^§^ A non-parametric test (Mann-Whitney test) was performed for between-group comparisons.*

*^†^IBA-1 positive microglia cells were counted in the basal ganglia.*

There were significant differences between the two groups regarding plasma total cholesterol, HDL-C, and LDL-C levels (*P* < 0.01, Table 1). Plasma urea and uric acid levels showed significant differences between the sham and BDL groups (*P* = 0.032 and *P* = 0.002, respectively), while plasma creatinine levels showed no significant differences between groups (*P* = 0.442, Table 1). Plasma 5-HT, IFN-γ, IL-1β, IL-6, IL-10, and TNF-α levels in the BDL group were significantly higher than those in the sham group (*P* < 0.01, Table 1). These biochemical measurements indicate liver impairments in the rats who underwent BDL.

### Micro-PET/CT Results

[^18^F]DPA-714 and [^18^F]PBR146 micro-PET/CT imaging was conducted on two consecutive days. Although the mean injected radioactivity of [^18^F]DPA-714 (279.3 ± 16.7 μci) and [^18^F]PBR146 (245.9 ± 19.2 μci) showed significant differences (*P* < 0.001), the injected radioactivities of [^18^F]DPA-714 and [^18^F]PBR146 in the sham group (270.9 ± 13.9 and 235.7 ± 19.4 μci, respectively) were not significantly different from the BDL group (286.5 ± 16.4 and 256.1 ± 13.4 μci; *P* = 0.094 and *P* = 0.060, respectively). All six rats in the sham group were subject to [^18^F]DPA-714 and [^18^F]PBR146 micro-PET/CT imaging. Seven rats in the BDL group were subject to [^18^F]DPA-714 micro-PET/CT imaging, while [^18^F]PBR146 micro-PET/CT scanning was conducted successfully on six BDL group rats, except that one rat died due to an anesthesia accident.

**FIGURE 1:**
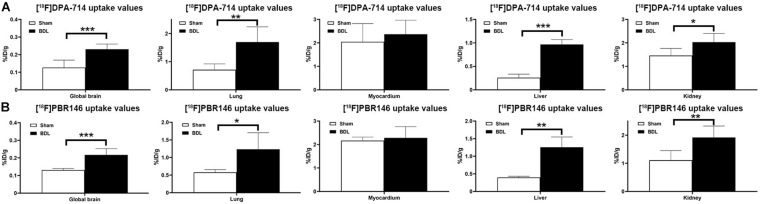
Histograms of uptake values in global brains and organs of sham and BDL rats. The [^18^F]DPA-714 uptake values in global brain and several organs **(A)** similar to that of [^18^F]PBR146 uptake values, **(B)** global brain, and most organs showed significantly differences between Sham and BDL rats except myocardium. BDL = bile duct ligation; *P < 0.05, **P < 0.01, and ***P < 0.001.

The average%ID/g values in the global brain of [^18^F]DPA-714 and [^18^F]PBR146 in the BDL rats were higher than those in the sham rats (*P* < 0.001, Figures 1A,B and Supplementary Table 1). There were significant differences in [^18^F]DPA-714 and [^18^F]PBR146 uptake values of the lung, liver, and kidney between the BDL and sham groups (*P* < 0.05), while there were no significant differences in the myocardium (*P* > 0.05, Figures 1A,B and Supplementary Table 1). Comparison results grouped by [^18^F]DPA-714 and [^18^F]PBR146 are shown in Table 2. Most organs showed no significant differences (*P* > 0.05), except that the livers of sham rats showed statistical differences between the two groups (*P* < 0.001, Table 2).

**TABLE 2:** Comparison of [^18^F]DPA-714 and [^18^F]PBR146 uptake values (%ID/g) in global brains and organs between sham and BDL rats.

Groups	[^18^F]DPA-714	[^18^F]PBR146	*P*
**Sham group**			
Global brain	0.127 ± 0.042	0.131 ± 0.009	0.800
Lung	0.715 ± 0.207	0.580 ± 0.076	0.165
Myocardium	2.047 ± 0.778	2.167 ± 0.151	0.719
Liver	0.259 ± 0.075	0.397 ± 0.036	0.002**
Kidney	1.460 ± 0.305	1.109 ± 0.342	0.125
**BDL group**			
Global brain	0.231 ± 0.029	0.217 ± 0.036	0.450
Lung	1.697 ± 0.542	1.234 ± 0.470	0.132
Myocardium	2.371 ± 0.596	2.283 ± 0.479	0.777
Liver	0.967 ± 0.105	1.256 ± 0.293	0.061
Kidney	2.033 ± 0.372	1.917 ± 0.407	0.616

**P < 0.01 was regarded as statistically significant. BDL = bile duct ligation.

**FIGURE 2:**
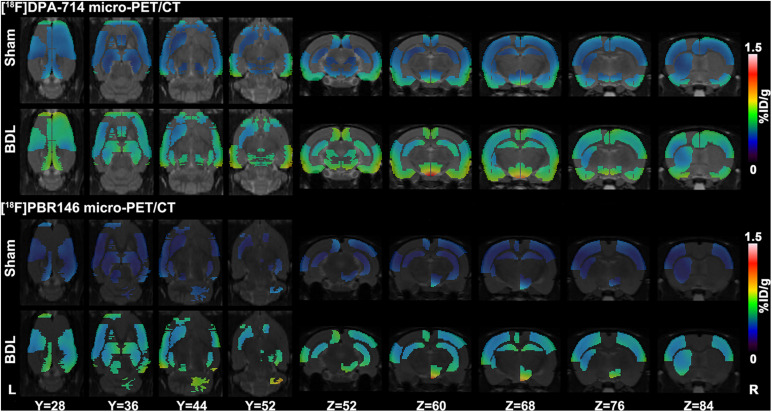
Representative [^18^F]DPA-714 and [^18^F]PBR146 micro-PET/CT images of sham and BDL rats. Brain regions, including the basal ganglia, cingulate cortex, auditory cortex, motor cortex, somatosensory cortex, hippocampus, thalamus, midbrain, and medulla, showed significant differences in average%ID/g values between the sham and BDL groups. The [^18^F]DPA-714 and [^18^F]PBR146 micro-PET/CT showed consistent results in these regions. BDL = bile duct ligation; L = left; R = right.

The comparison of [^18^F]DPA-714 and [^18^F]PBR146 uptake values in regional brain areas showed consistent results (Supplementary Tables 2, 3). Significant differences among brain regions between the sham and BDL groups were mainly located in the basal ganglia, cingulate cortex, auditory cortex, motor cortex, somatosensory cortex, hippocampus, thalamus, midbrain, and medulla (*P* < 0.05, Figure 2). The average%ID/g values of the BDL group were higher than the sham group in the brain regions without statistical differences (*P* > 0.05, Supplementary Tables 2, 3).

### Histopathological and Immunohistochemical Characterizations

**FIGURE 3:**
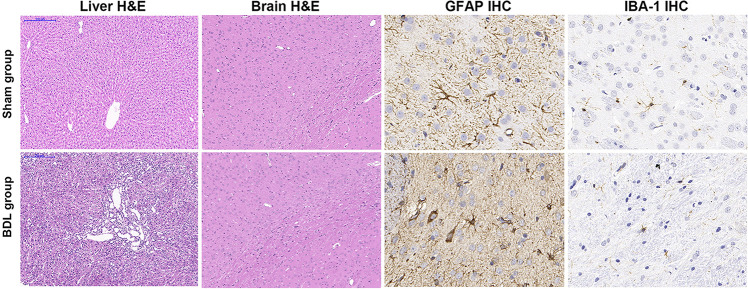
Representative H&E staining and IHC findings in sham and BDL rats. Liver H&E staining (100 × magnification) showed normal hepatic histology in the sham group. Staining in the BDL group showed destroyed hepatic cords, expanded bile duct, and inflammatory infiltration. H&E staining (100×) of brain tissue showed no significant differences between the sham and BDL groups. GFAP IHC micrographs of microglia in the basal ganglia were similar to IBA-1 IHC findings (400×). Microglial cells in the sham rats showed ramified shapes (resting microglia), while microglial cells in the BDL rats showed amoeboid shapes (activated microglia) in the basal ganglia. H&E = hematoxylin-eosin; IHC = immunohistochemistry; BDL = bile duct ligation; GFAP = glial fibrillary acidic protein; IBA–1 = ionized calcium-binding adapter molecule 1.

Liver weights in the sham group (17.87 ± 1.76 g) were significantly lower than the BDL group (24.71 ± 6.44 g, *P* = 0.048), suggesting that the livers of rats in the BDL group showed compensatory hyperplasia. Liver H&E staining in the sham group showed normal hepatic structure, while staining in the BDL group revealed biliary cirrhosis with destroyed hepatic cords, expanded bile ducts, and inflammatory infiltration. Additionally, the jejunum, ileum, and colon tissues showed normal intestinal structures in the sham and BDL groups (Figure 3). Brain tissue after H&E staining showed no significant differences between the sham group and BDL group, while histopathological changes were observed during GFAP and IBA-1 immunohistochemical analysis of brain microglia. In particular, microglial cells in the sham rats showed ramified shapes (resting microglia), while they showed amoeboid shapes (activated microglia) in the BDL rats (Figure 3). The sham group showed significantly lower amounts of IBA-1 immune reactive microglia in the basal ganglia than BDL rats (*P* = 0.006, Table 1).

### Relationship Between Behavioral Results and Micro-PET/CT Results

Duration of time spent on the rotarod and number of crossovers during the motor activity test showed no correlation with [^18^F]DPA-714 uptake values (*P* > 0.05), while the beam crossing time showed a positive correlation with [^18^F]DPA-714 uptake values in the global brain (*r* = 0.571, *P* = 0.042), including structures such as the bilateral accumbens, striatum, auditory cortex, cingulate cortex, insular cortex, medial prefrontal cortex, orbitofrontal cortex, visual cortex, olfactory, right amygdala, motor cortex, retrosplenial cortex, hippocampus anterodorsal, hippocampus posterior, hypothalamus, and colliculus superior (*P* < 0.05, Supplementary Table 4).

Beam walking cross time showed no correlation with [^18^F]PBR146 uptake values, and the behavioral results showed no correlation with [^18^F]PBR146 uptake values in the global brain (*P* > 0.05, Supplementary Table 5). Duration of time spent on the rotarod showed a negative correlation with [^18^F]PBR146 uptake values in the bilateral auditory cortex, cingulate cortex, entorhinal cortex, motor cortex, hypothalamus, left accumbens, medial prefrontal cortex, retrosplenial cortex, hippocampus antero dorsal, right amygdala, frontal association cortex, somatosensory cortex, hippocampus posterior, and olfactory (*P* < 0.05, Supplementary Table 5). The number of crossovers during the motor activity test showed a negative correlation with [^18^F]PBR146 uptake values in the bilateral entorhinal cortex, insular cortex, olfactory, left amygdala, hypothalamus, and right accumbens (*P* < 0.05, Supplementary Table 5).

## Discussion

The pathogenesis of HE has received increased attention in neuroinflammation research in recent years, which has provided new insight into non-invasive imaging methods for HE monitoring (Butterworth, 2013; Alam et al., 2017). Higher TSPO expression in activated microglia during neuroinflammation provides a novel target for radiotracers in PET imaging in order to identify neuroinflammation in various central nervous system diseases (Alam et al., 2017). In the present study, [^18^F]PBR146 and [^18^F]DPA-714 uptake values were measured in the brains and organs of rats modeling chronic HE compared to control model rats. It was found that both [^18^F]DPA-714 and [^18^F]PBR146 uptake values were useful for neuroinflammation imaging in BDL rats. Abnormal brain regions were associated with cognitive impairment, suggesting that neuroinflammation is involved in the disease progression of HE.

The International Society on Hepatic Encephalopathy and Nitrogen Metabolism recommended BDL as the experimental rat model of chronic HE, which induces biliary cirrhosis with hyperammonemia and jaundice after more than 28 days post-surgery (Butterworth et al., 2009). The mortality of BDL rats in this study was 33% (4/12), mainly due to liver failure with infection. The serum ammonia level of BDL rats was significantly higher than that of Sham rats, and the liver function, such as bilirubin levels, ALT, and AST levels, were significantly increased in BDL rats. The renal function indicators also showed differences between the two groups, but these alterations were slight compared with liver injury in BDL rats. Liver tissue H&E staining results also showed chronic liver injury, and the chronic HE BDL rats showed decreased motor activity and coordination deficits in behavioral tests. Previous studies reported that [^18^F]DPA-714 shows greater promise for modeling acute or chronic HE (Kong et al., 2016; Luo et al., 2018). The peripheral organs uptake values were similar between [^18^F]DPA-714 and [^18^F]PBR146, all radiotracers uptake values in the lungs, liver, and kidneys of the BDL group were higher than those in the sham group, and plasma inflammatory factors were also significantly increased in BDL rats, suggesting that the initial systemic inflammatory response syndrome because of cytokine storm was related to end-stage liver failure and might serve as a prerequisite for neuroinflammation in chronic HE (Kong et al., 2016; Sarin and Choudhury, 2018). It is noteworthy that the [^18^F]PBR146 uptake values in the liver were significantly higher than [18F]DPA-714, this might suggest that [^18^F]PBR146 could be further applied widely in chronic liver failure animal models, requiring more studies in the future. [^18^F]PBR146 was first reported by Fookes et al. (2008), but it has not yet been applied to HE models. However, it was found that [^18^F]PBR146 was not inferior to [^18^F]DPA-714 regarding its neuroinflammation imaging ability in chronic HE rats. The uptake values in the liver and global brain were significantly different between the BDL and sham rats in this study.

In addition, the regional brain area results were similar between [^18^F]PBR146 and [^18^F]DPA-714 micro-PET/CT imaging in structures including the basal ganglia, cingulate cortex, auditory cortex, motor cortex, somatosensory cortex, and hippocampus. The brain regions with statistical significance were inconsistent between the two radiotracers, which might be because of the statistical errors in the controlled range. Agusti et al. (2014) found that [^3^H]-PK11195 uptake by the striatum, frontal cortex, parietal cortex, thalamus, and hippocampus were statistically increased in a minimal HE rat model due to portacaval shunt compared to sham rats, which was consistent with this study. These results were also similar to another previous study (Kong et al., 2016). It was found that [^18^F]DPA-714 uptake values in the global brain were positively correlated with beam walking cross time and that [^18^F]DPA-714 uptake values in regional brain areas (in this case, the basal ganglia, cingulate cortex, auditory cortex, motor cortex, somatosensory cortex, and hippocampus) were positively correlated with beam walking cross time. However, beam cross time showed no correlation with [^18^F]PBR146 uptake values. It was found that the duration of time on the rotarod and the number of crossovers during the motor activity test showed a negative correlation with [^18^F]PBR146 uptake values in the basal ganglia, cingulate cortex, entorhinal cortex, motor cortex, somatosensory cortex, and hippocampus.

The basal ganglia are associated with motor, cognitive, and affective functions (Bostan and Strick, 2018). The cingulate cortex is associated with rewards to actions involved in emotion (Rolls, 2019). The auditory cortex is essential for analyzing the identity and behavioral importance of tones paired with emotional events (Concina et al., 2019). The hippocampal-entorhinal circuits are crucial to episodic memory and require the encoding of time and binding to events (Tsao et al., 2018). Finally, the motor cortex is important for motor skill learning (Papale and Hooks, 2018). [^18^F]DPA-714 and [^18^F]PBR146 uptake values in these brain regions showed correlations with behavioral alterations, suggesting that neuroinflammation may contribute to cognitive dysfunction in a rat model of chronic HE (Kong et al., 2016).

This study has some limitations that need to be considered. First, the sample size of each group may have influenced the accuracy of statistical results. Second, the behavioral studies mainly evaluated motor and emotional parameters; thus, the learning abilities of the rats should be examined in a further investigation (i.e., by performing a water maze or Y maze test). Third, the study lacked TSPO immunohistochemistry staining, immunofluorescent staining, and autoradiography to exclude potential imaging affected factors. Fourth, indicators of TSPO quantification and suitable analytical models to explain either radiotracer uptake values in the brain or behavior or biochemical examination results. Fifth, further studies are needed to investigate the effects of different treatments on chronic HE.

In conclusion, both [^18^F]PBR146 and [^18^F]DPA-714 can be used for TSPO imaging of chronic HE in rats. These two radiotracers may help quantitatively evaluate the neuroinflammation load and distribution of different brain regions correlated with partial behavioral alterations. Thus, [^18^F]PBR146 and [^18^F]DPA-714 may non-invasively monitor neuroinflammation, which will be useful in future investigations of treatment efficacy in neuroinflammation of BDL-induced chronic HE.

## Data Availability Statement

The raw data supporting the conclusions of this article will be made available by the authors, without undue reservation.

## Ethics Statement

The animal study was reviewed and approved by The Animal Experimental Ethics Committee of Jinling Hospital, Medical School of Nanjing University.

## Author Contributions

GL and LZ conceived and designed the study. XK, YW, and SL contributed to the literatures search. XK and GY completed the experiment and the writing and revision of this manuscript. All authors approved it for publication.

## Conflict of Interest

The authors declare that the research was conducted in the absence of any commercial or financial relationships that could be construed as a potential conflict of interest.

## Publisher’s Note

All claims expressed in this article are solely those of the authors and do not necessarily represent those of their affiliated organizations, or those of the publisher, the editors and the reviewers. Any product that may be evaluated in this article, or claim that may be made by its manufacturer, is not guaranteed or endorsed by the publisher.
