# Supplementary material for: [18F]PBR146 and [18F]DPA-714 in vivo Imaging of Neuroinflammation in Chronic Hepatic Encephalopathy Rats
Source: Front Neurosci. 2021 Aug 16;15:678144. doi: 10.3389/fnins.2021.678144 (PMC8415356; doi:10.3389/fnins.2021.678144)
Supplement: Supplementary file 1 [file Data_Sheet_1.docx]

# Supplementary materials

# Materials and Methods

## Bile duct ligation (BDL)

Rats were general anesthetized by using 10% chloral hydrate solution injected intraperitoneally in 0.4 ml/100 g of body weight. Under sterile condition, a midline incision was made at upper abdomen, and the common bile duct was segregated, doubly ligated below the bifurcation close to the liver, and served between the two ligatures (Rodrigo et al., 2010; Kong et al., 2016).

## Behavior studies

**-Rotarod test.** An accelerating rotarod apparatus (Ugo Basile, Comerio, Italy) was used to evaluate the motor coordination of rats. Two consecutive days before testing, each rat was placed on the rotating drum for 5 minutes with a constant speed of 6 revolutions per minute (RPM) twice a day for acclimatization. In the test, the rotarod speed increased from 6 to 40 RPM over 5 minutes. The time at which each rat fell off the rotarod was recorded in seconds with a maximum of 600 seconds (Agusti et al., 2011; Kong et al., 2016). Three trials were performed to average the testing values with 15 minutes interval time.

**-Beam walking test.** This test was used to evaluate the rats’ motor coordination ability to pass through a narrow beam to reach a dark box (Agusti et al., 2011; Jover et al., 2006; Kong et al., 2016). A homemade wooden square beam (120 cm × 2 cm × 4 cm) was placed to a height of 0.5 m above the ground with a white light illuminating the beginning to encourage the rats to walk through the beam. In the test, the time to cross the beam and the forelimb and hindlimb foot faults numbers were manually recorded. The fault was defined as any foot slip off the beam top surface or any limb fall on the side of beam. For adaptive training to the beam and the surrounding condition, four trials were performed before recording the results, and the interval time between trials was at least 5 minutes.

**-Motor activity.** Rats were placed individually in an open-field activity chamber (50 cm long, 35 cm wide, and 20 cm high) with sawdust at the bottom. The crossovers through a line dividing the cages into two compartments were counted locomotor activity, and the number of rearings was measured as vertical activity. The motor activity was recorded for 15 minutes. This test could reflect the motor activity alterations of rats (Kong et al., 2016).

## Radiosynthesis of ligands

The synthesis of [^18^F]DPA-714 is relatively complicated as previously reported (Kong et al., 2016; Lavisse et al., 2015; Luo et al., 2018). No-carrier-added aqueous [^18^F]fluoride ion radionuclide was obtained from a cyclotron by proton irradiation of 95% enriched ^18^O-H_2_O via the ^18^O (p, n) [^18^F] nuclear reaction. The [^18^F]Fluoride, which was trapped and eluted from an anion-exchange resin of ^18^O-H_2_O, was transferred to the reaction vessel with the solution containing H_2_O/CH_3_CN, K_2_CO_3_, and Kryptofix-222. Then, the reaction mixture was added to aliquots of acetonitrile and evaporated. The dry [^18^F]labeled K–F–Kryptofix-222 complex was added to the mixture of toluene-4-sulfonic acid 2-[4-(3-diethylcarbamoylmethyl-5,7-dimethyl-pyrazolo-[1,5-a]pyrimidin-2-yl)-phenoxy]-ethyl ester and acetonitrile, and heated to 80°C for about 10 min. After the CH_3_CN was evaporated off, the crude product was dissolved in CH_3_COONH_4_ and purified by semi-preparative reversed-phase high performance liquid chromatography (HPLC) column which was eluted with CH_3_COONH_4_/CH_3_CN (65/35% v/v). Then, [^18^F]DPA-714 was washed out with isotonic saline containing 5% ethanol and filtered using sterile technique. The final product was analyzed by HPLC quality control of [^18^F]DPA-714. [^18^F]DPA-714 has similar retention time of absorption peak in HPLC with [^19^F]DPA-714, but the peak magnitude was different (**Supplementary figure S2**). Synthetic time was 60-70 min, and radiochemical yield of [^18^F]DPA-714 was of 45±3.0%. Radiochemical purity was > 95% and the specific activity ranged from 61.4 to 73.5 GBq/μmol.

The synthetic route of [^18^F]PBR146 [N,N-diethyl-2-(2-(4-(3-[^18^F]fluoropropoxy)phenyl)-5,7-dimethylpyrazolo[1,5-a]pyrimidin-3-yl)acetamide] was illustrated in **Supplementary figure S3A** referring to previous study (Fookes et al., 2008). **Step 1:** Methyl 4-hydroxybenzoate (9.615g, 63.2mmol) and anhydrous potassium carbonate (30.570g, 221mmol) was slowly treated with 150mL acetone and 2-bromopropane (11.660g, 94.8mmol). And the mixture was sealed at 85 °C for 72 h followed by concentrated and separated by chromatography column (PE:EA=20:1), then obtained 11.627g colourless liquid (Compound 1) with yield 94.7%. ^1^H NMR (300 MHz, CDCl_3_) δ 7.97 (2H, d, J=9.0 Hz), 6.88 (2H, d, J=8.9 Hz), 4.66-4.58 (1H, sep), 3.87 (3H, s), 1.35 (6H, d, J=6.0Hz), LC-MS: calculated for C_11_H_14_O_3_, 194.23, found [M+H] 195.1. **Step 2:** Compound 1 (11.627g, 59.86mmol) in methylbenzene (60 mL) was cooled to 0 °C followed by 60% sodium hydride (4.789g, 119.72mmol), heated to 80 °C stirred for 10 min, and then dropped with acetonitrile (12.287g, 299.68mmol) to methylbenzene heated to 85 °C for 16-18 h. After cooling, the solution was added with n-hexane to stir and filter, and the filtrate was dissolved in 500mL ice water followed by extracting by ethyl acetate (250mL*2), then successively dried, concentrated and separated by chromatography column (PE:EA=10:1) to give a white solid (Compound 2, 9.791g, 80.5%). ^1^H NMR (300 MHz, CDCl_3_) δ 7.88 (2H, d, J=8.9 Hz), 6.93 (2H, d, J=8.9 Hz), 4.71-4.63 (1H, sep), 4.01 (2H, s), 1.38 (6H, d, J=6.1 Hz), LC-MS: calculated for C_12_H_13_NO_2_, 203.24, found [M+H] 204.1. **Step 3:** Sodium hydroxide (0.455g, 10.94mmol) and ethylalcohol (80%, 80mL) were stirred for 15 min at room temperature, then the mixture was added sodiumiodide (4.441g, 29.63mmol), Compound 2 (2.180g, 9.94mmol), and N,N-diethylacetamide (1.488g, 9.94mmol) successively at 20-25 °C for 16-18 h followed by concentrated and separated by chromatography column (n-Hexane:EA=10:1~8:1~5:1~3:1) to afford an oily product (Compound 3, 2.467g, 72.7%). ^1^H NMR (300 MHz, CDCl_3_) δ 8.02 (2H, d, J=9.0 Hz), 6.95 (2H, d, J=9.0 Hz), 5.00 (1H, dd, J=9.2, 4.6 Hz), 4.71-4.63 (1H, sep), 3.44-3.38 (4H, m), 2.85 (1H, dd, J=16.1, 4.6 Hz), 1.38-1.36 (6H, d, J=6.0 Hz), 1.29-1.23 (3H, t, J=7.1 Hz), 1.11-1.06 (3H, t, J=7.1Hz), LC-MS: calculated for C_18_H_24_N_2_O_3_, 316.40, found [M+H] 317.2. **Step 4:** Compound 3 (2.460g, 7.77mmol) and ethylalcohol (80%, 50mL) were added with 80% hydrazine hydrate (0.973g, 15.55mmol) and acetic acid (0.747g, 12.44mmol) boiled to 100 °C with reflux reaction for 4 h, then concentrated up to dryness. After cooling, the solution was added with 50mL 2N hydrochloric acid and 50mL diethyl ether stirred for 20 min separated out off-white solid, then the filtrate was washed with diethyl ether and added with 100 mL water followed by basifing to pH 12 with 50 mL 1N sodium hydroxide and extracting by dichloromethane (100mL*2) to dryness, and collected 1.888g off-white solid (Compound 4, 73.5%). ^1^H NMR (300 MHz, CDCl_3_) δ 7.28 (2H, d, J=8.7 Hz), 6.94 (2H, d, J=8.7 Hz), 4.63-4.55 (1H, sep), 3.50 (2H, s), 3.33 (2H, q, J=7.2 Hz), 3.16 (2H, q, J=7.2 Hz), 1.36 (6H, d, J=6.1 Hz), 1.08 (3H, t, J=7.1 Hz), 0.92 (3H, t, J=7.1 Hz); LC-MS: calculated for C_18_H_26_N_4_O_2_, 330.43, found [M+H] 331.1. **Step 5:** Compound 4 (1.888g, 5.7mmol) and ethylalcohol (80%, 30mL) were added with acetylacetone (0.572g, 5.7mmol) for reflux reaction at 90 °C for 16-18 h. After concentration, the mixture was recrystallized with isopropanol to obtain a white spumescence solid (Compound 5, 1.530g, 67.8%). ^1^H NMR (300 MHz, CDCl_3_) δ 7.75 (2H, d, J=8.8 Hz), 6.96 (2H, d, J=8.8 Hz), 6.49 (1H, s), 4.64-4.56 (1H, sep), 3.91 (2H, s), 3.50 (2H, q, J=7.2 Hz), 3.41 (2H, q, J=7.0 Hz), 2.74 (3H, s), 2.53 (3H, s), 1.36-1.34 (6H, d, J=6.0 Hz), 1.20 (3H, t, J=7.1 Hz), 1.11 (3H, t, J=7.1 Hz), LC-MS: calculated for C_23_H_30_N_4_O_2_, 394.52, found [M+H] 395.2. **Step 6:** Compound 5 (5.268g, 13.4mmol) and 100 mL dichloromethane were added with anhydrous aluminum chloride (5.876g, 44.1mmol) in ice bath followed by reflux reaction at 30 °C for 2 h. After cooling to 0 °C, the mixture was added with saturated ammonium chloride solution and stirred for 30 min to quench, and then extracted by dichloromethane (100mL*2) to dryness, and separated by chromatography column (DCM:MeOH=30:1) to give a white solid (Compound 6, 3.627g, 77.1%). ^1^H NMR (300 MHz, CDCl_3_) δ 7.65 (2H, d, J=8.6 Hz), 7.30 (1H, brs), 6.82 (2H, d, J=8.6 Hz), 6.49 (1H, s), 3.96 (2H, s), 3.51 (2H, q, J=7.1 Hz), 3.38(2H, q, J=7.2 Hz), 2.73 (3H, s), 2.53 (3H, s), 1.15 (3H, t, J=7.1 Hz), 1.08 (3H, t, J=7.1 Hz), LC-MS: calculated for C_20_H_24_N_4_O_2_, 352.44, found [M+H] 353.2. **Step 7:** Compound 6 (0.300g, 0.85mmol) in anhydrous potassium carbonate (0.413g, 2.99mmol) was slowly treated with acetonitrile (10mL) followed by F(CH_2_)3OTS (0.198g, 0.85mmol) and the mixture sealed at 80 °C for 16-18 h. The reaction solution was poured into water extracted with ethyl acetate (80mL*2), then the concentrate was separated by chromatography column (DCM:MeOH=100:1~50:1) followed by ether evaporated, further purified by chromatography column (dichloromethane:methanol=100:1~50:1~30:1) afford 0.342g off-white solid. Eventually, the solid was recrystallized from ethyl acetate and n-hexane to give the PBR146 (Compound 7) as white solid (0.221g, 63.0%). ^1^H NMR (300 MHz, CDCl_3_) δ 7.75 (2H, d, J=8.7 Hz), 6.98(2H, d, J=8.7 Hz), 6.50 (1H, s), 4.66 (2H, dt, J=47.1, 5.8 Hz), 4.15 (2H, t, J=6.1 Hz), 3.91 (2H, s), 3.51(2H, q, J=7.2 Hz), 3.41 (2H, q, J=7.1 Hz), 2.74 (3H, s), 2.53 (3H, s), 2.21 (2H, dm, J=26.0 Hz), 1.20 (3H, t, J=7.1Hz), 1.11 (3H, t, J=7.1 Hz), LC-MS: calculated for C_23_H_29_FN_4_O_2_, 412.51, found [M+H] 413.4. In addition, the [^18^F]fluoride labeled procedure to PBR146 was similar with [^18^F]DPA-714. The final product was analyzed by HPLC quality control of [^18^F]PBR146. [^18^F]PBR146has similar retention time of absorption peak in HPLC with [^19^F]PBR146, but the peak magnitude was different (**Supplementary figure S3B and C**). Synthetic time was 60 min, and radiochemical yield of [^18^F]PBR146 was of 63.0%. Radiochemical purity was > 95% and the specific activity ranged from 85.7 to 104.9 GBq/μmol.

# References

Agusti, A., Cauli, O., Rodrigo, R., Llansola, M., Hernández-Rabaza, V., and Felipo, V. (2011). p38 MAP kinase is a therapeutic target for hepatic encephalopathy in rats with portacaval shunts. Gut. 60, 1572-1579. doi: 10.1136/gut.2010.236083

Fookes, C. J., Pham, T. Q., Mattner, F., Greguric, I., Loc'h, C., Liu, X., et al. (2008). Synthesis and biological evaluation of substituted [^18^F]imidazo[1,2-a]pyridines and [^18^F]pyrazolo[1,5-a]pyrimidines for the study of the peripheral benzodiazepine receptor using positron emission tomography. *J Med Chem.* 51, 3700-3712. doi: 10.1021/jm7014556

Jover, R., Rodrigo, R., Felipo, V., Insausti, R., Sáez-Valero, J., García-Ayllón, M. S., et al. (2006). Brain edema and inflammatory activation in bile duct ligated rats with diet-induced hyperammonemia: A model of hepatic encephalopathy in cirrhosis. *Hepatology.* 43, 1257-1266. doi: 10.1002/hep.21180

Kong, X., Luo, S., Wu, J. R., Wu, S., De Cecco, C. N., Schoepf, U. J., et al. (2016). ^18^F-DPA-714 PET imaging for detecting neuroinflammation in rats with chronic hepatic encephalopathy. *Theranostics.* 6, 1220-1231. doi: 10.7150/thno.15362

Lavisse, S., Inoue, K., Jan, C., Peyronneau, M. A., Petit, F., Goutal, S., et al. (2015). [^18^F]DPA-714 PET imaging of translocator protein TSPO (18 kDa) in the normal and excitotoxically-lesioned nonhuman primate brain. *Eur J Nucl Med Mol Imaging.* 42, 478-494. doi: 10.1007/s00259-014-2962-9

Luo, S., Kong, X., Wu, J. R., Wang, C.Y., Tian, Y., Zheng, G., et al. (2018). Neuroinflammation in acute hepatic encephalopathy rats: imaging and therapeutic effectiveness evaluation using ^11^C-PK11195 and ^18^F-DPA-714 micro-positron emission tomography. *Metab Brain Dis.* 33, 1733-1742. doi: 10.1007/s11011-018-0282-7

Rodrigo, R., Cauli, O., Gomez-Pinedo, U., Agusti, A., Hernandez-Rabaza, V., Garcia-Verdugo, J. M., et al. (2010). Hyperammonemia induces neuroinflammation that contributes to cognitive impairment in rats with hepatic encephalopathy. *Gastroenterology.* 139, 675-684. doi: 10.1053/j.gastro.2010.03.040

# Supplementary figures and figure legends


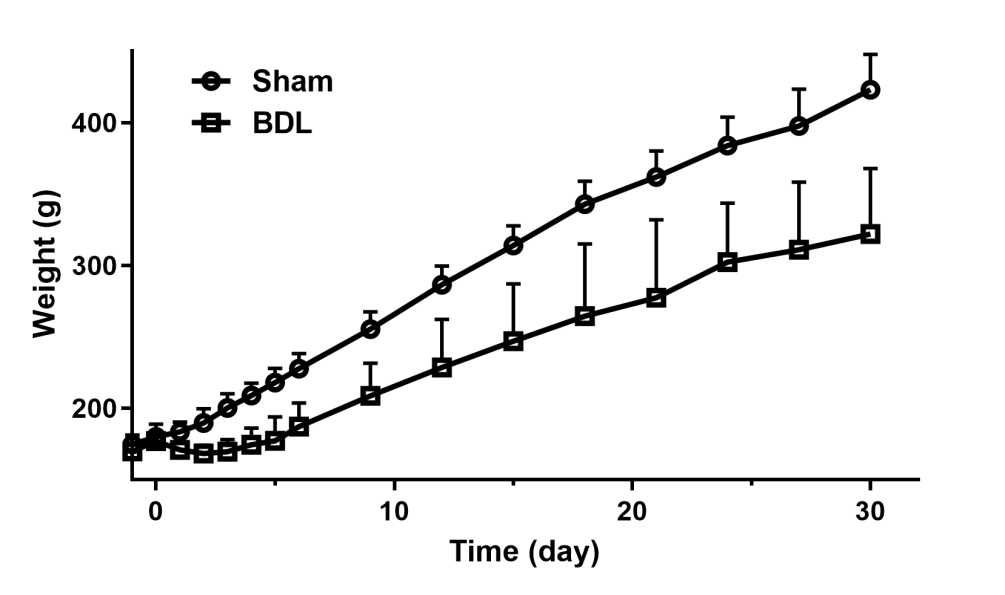


## Supplementary figure S1. Weight change curves of sham and BDL rats.

Weights of sham-operated rats were generally higher than BDL-operated rats during the study. BDL = bile duct ligation


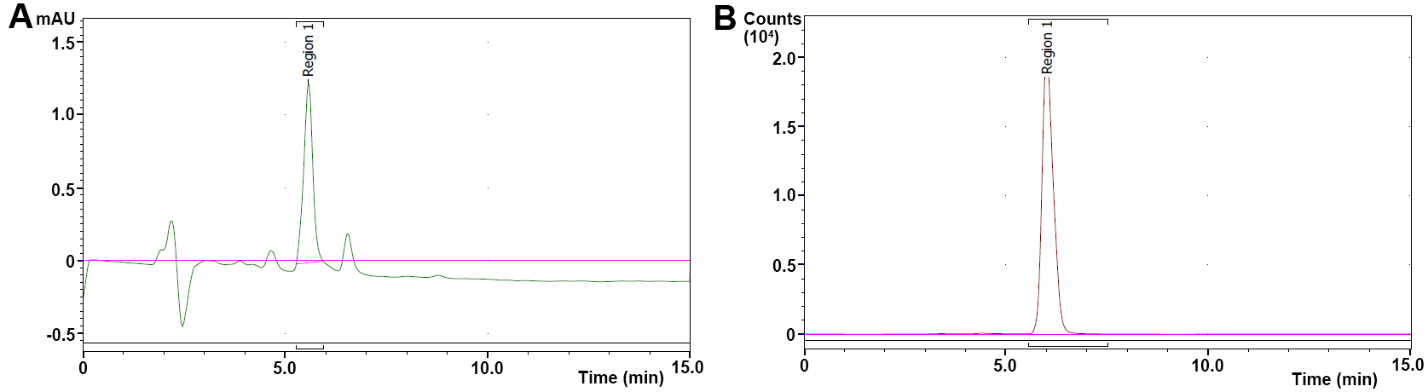


## Supplementary figure S2. The HPLC results of [^19^F]DPA-714 and [^18^F]DPA-714.

The retention time of absorption peak of [^19^F]DPA-714 and [^18^F]DPA-714 were about 5.34 min **(A)** and 6.01 min **(B)** respectively, the peak magnitude was different.


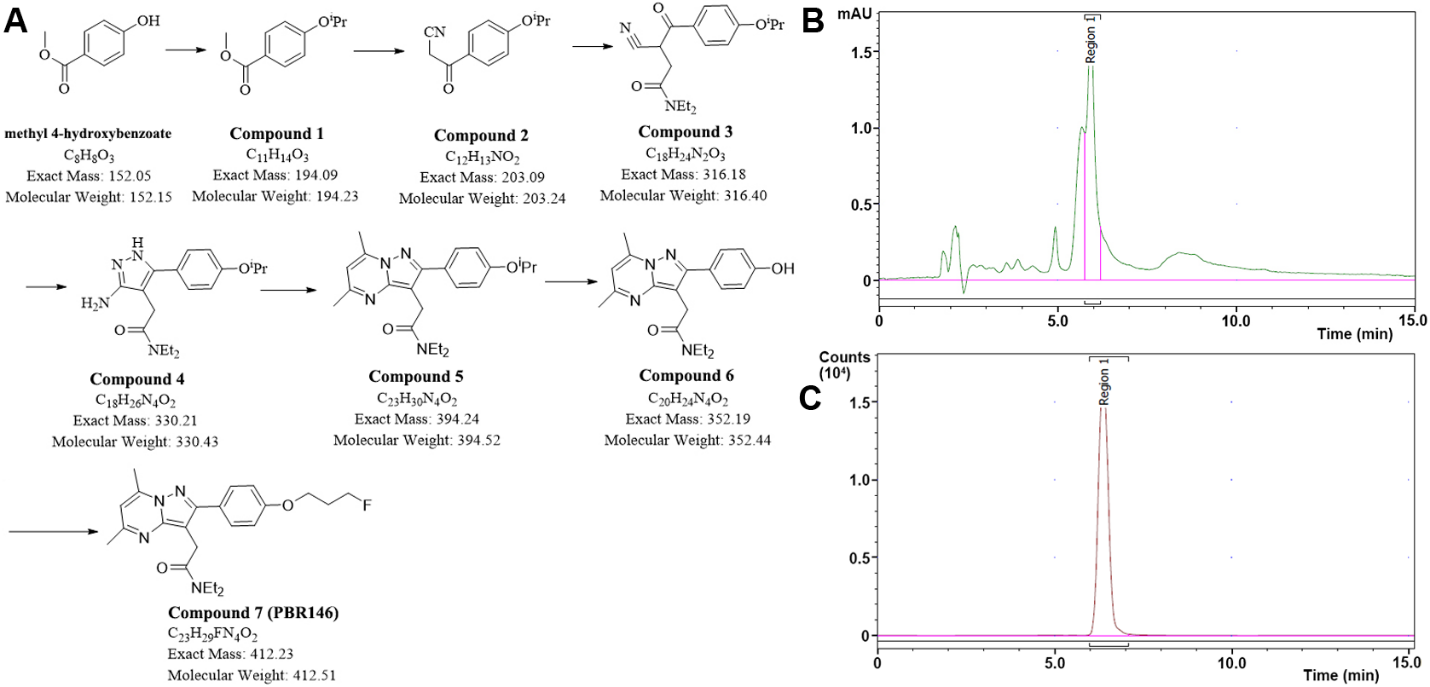


## Supplementary figure S3. The synthetic route of PBR146 compound and HPLC results of [^19^F]PBR146 and [^18^F]PBR146.

**(A)** showed the synthetic route of PBR146 compound. The retention time of absorption peak of [^19^F]PBR146 and [^18^F]PBR146 was about 5.90 min **(B)** and 6.24 min **(C)** respectively, the peak magnitude was different. HPLC = high performance liquid chromatography.
