# Supplementary material for: [18F]PBR146 and [18F]DPA-714 in vivo Imaging of Neuroinflammation in Chronic Hepatic Encephalopathy Rats
Source: Front Neurosci. 2021 Aug 16;15:678144. doi: 10.3389/fnins.2021.678144 (PMC8415356; doi:10.3389/fnins.2021.678144)
Supplement: Supplementary file 2 [file Table_1.docx]

## Supplementary table S1. Comparison of [^18^F]DPA-714 and [^18^F]PBR146 uptake values in global brain and several organs between Sham and BDL groups (%ID/g)

| **Organs** | **Sham group** | **BDL group** | ***P*** |
| --- | --- | --- | --- |
| **[^18^F]DPA-714** |  |  |  |
| Global brain | 0.127±0.042 | 0.231±0.029 | <0.001*** |
| Lung | 0.715±0.207 | 1.697±0.542 | 0.001** |
| Myocardium | 2.047±0.778 | 2.371±0.596 | 0.413 |
| Liver | 0.259±0.075 | 0.967±0.105 | <0.001*** |
| Kidney | 1.460±0.305 | 2.033±0.372 | 0.022* |
| **[^18^F]PBR146** |  |  |  |
| Global brain | 0.131±0.009 | 0.217±0.036 | <0.001*** |
| Lung | 0.580±0.076 | 1.234±0.470 | 0.018* |
| Myocardium | 2.167±0.151 | 2.283±0.479 | 0.590 |
| Liver | 0.397±0.036 | 1.256±0.293 | 0.001** |
| Kidney | 1.109±0.342 | 1.917±0.407 | 0.007** |

Note: Values are the mean ± standard deviation. Six rats in Sham group and seven rats in BDL group were performed [^18^F]DPA-714 micro-PET/CT imaging, and six rats in each group were performed [^18^F]PBR146 micro-PET/CT imaging.

* *P*<0.05, ***P*<0.01, and ****P*<0.001 were regarded as statistically significant. BDL = bile duct ligation.
