# Supplementary material for: [18F]PBR146 and [18F]DPA-714 in vivo Imaging of Neuroinflammation in Chronic Hepatic Encephalopathy Rats
Source: Front Neurosci. 2021 Aug 16;15:678144. doi: 10.3389/fnins.2021.678144 (PMC8415356; doi:10.3389/fnins.2021.678144)
Supplement: Supplementary file 3 [file Table_2.docx]

## Supplementary table S2. Comparison of [^18^F]DPA-714 uptake values in regional brain between Sham and BDL groups (%ID/g)

| **Brain Regions** | **Sham (n=6)** | **BDL (n=7)** | ***P*** |
| --- | --- | --- | --- |
| Accumbens_L | 0.108±0.048 | 0.165±0.027 | 0.021* |
| Accumbens_R | 0.080±0.032 | 0.142±0.062 | 0.045* |
| Amygdala_L | 0.149±0.049 | 0.211±0.045 | 0.036* |
| Amygdala_R | 0.142±0.051 | 0.214±0.059 | 0.040* |
| Striatum_L | 0.087±0.034 | 0.146±0.044 | 0.025* |
| Striatum_R | 0.114±0.047 | 0.155±0.041 | 0.119 |
| Auditory Cortex_L | 0.128±0.049 | 0.200±0.049 | 0.023* |
| Auditory Cortex_R | 0.138±0.041 | 0.202±0.055 | 0.038* |
| Cingulate Cortex_L | 0.085±0.036 | 0.161±0.051 | 0.011* |
| Cingulate Cortex_R | 0.085±0.033 | 0.161±0.045 | 0.006** |
| Entorhinal Cortex_L | 0.166±0.063 | 0.254±0.050 | 0.017* |
| Entorhinal Cortex_R | 0.167±0.059 | 0.247±0.062 | 0.037* |
| Frontal Association Cortex_L | 0.119±0.045 | 0.178±0.070 | 0.101 |
| Frontal Association Cortex_R | 0.108±0.039 | 0.179±0.058 | 0.027* |
| Insular Cortex_L | 0.106±0.038 | 0.194±0.057 | 0.008** |
| Insular Cortex_R | 0.111±0.044 | 0.181±0.038 | 0.011* |
| Medial Prefrontal Cortex_L | 0.113±0.047 | 0.169±0.058 | 0.086 |
| Medial Prefrontal Cortex_R | 0.104±0.032 | 0.169±0.049 | 0.019* |
| Motor Cortex_L | 0.094±0.037 | 0.148±0.054 | 0.062 |
| Motor Cortex_R | 0.098±0.036 | 0.165±0.055 | 0.028* |
| Orbitofrontal Cortex_L | 0.120±0.040 | 0.191±0.058 | 0.028* |
| Orbitofrontal Cortex_R | 0.119±0.042 | 0.173±0.034 | 0.027* |
| Para Cortex_L | 0.082±0.028 | 0.113±0.038 | 0.133 |
| Para Cortex_R | 0.093±0.036 | 0.119±0.036 | 0.220 |
| Retrosplenial Cortex_L | 0.136±0.063 | 0.215±0.063 | 0.044* |
| Retrosplenial Cortex_R | 0.137±0.061 | 0.235±0.072 | 0.023* |
| Somatosensory Cortex_L | 0.095±0.031 | 0.143±0.036 | 0.027* |
| Somatosensory Cortex_R | 0.100±0.034 | 0.160±0.047 | 0.024* |
| Visual Cortex_L | 0.119±0.047 | 0.163±0.043 | 0.103 |
| Visual Cortex_R | 0.122±0.043 | 0.177±0.048 | 0.055 |
| Hippocampus Antero Dorsal_L | 0.097±0.030 | 0.174±0.062 | 0.018* |
| Hippocampus Antero Dorsal_R | 0.120±0.041 | 0.203±0.061 | 0.017* |
| Hippocampus Posterior_L | 0.137±0.051 | 0.189±0.053 | 0.099 |
| Hippocampus Posterior_R | 0.126±0.061 | 0.191±0.051 | 0.061 |
| Hypothalamus_L | 0.134±0.061 | 0.229±0.074 | 0.029* |
| Hypothalamus_R | 0.121±0.045 | 0.204±0.070 | 0.030* |
| Olfactory_L | 0.154±0.058 | 0.241±0.069 | 0.032* |
| Olfactory_R | 0.137±0.050 | 0.212±0.047 | 0.018* |
| Colliculus Superior_L | 0.089±0.043 | 0.180±0.066 | 0.013* |
| Colliculus Superior_R | 0.104±0.043 | 0.194±0.059 | 0.010* |
| Midbrain_L | 0.089±0.035 | 0.172±0.068 | 0.020* |
| Midbrain_R | 0.093±0.040 | 0.185±0.074 | 0.017* |
| Ventral Tegmental Area_L | 0.104±0.038 | 0.197±0.068 | 0.012* |
| Ventral Tegmental Area_R | 0.108±0.045 | 0.198±0.057 | 0.010* |
| Cerebellum-Grey_L | 0.179±0.064 | 0.253±0.069 | 0.071 |
| Cerebellum-Grey_R | 0.165±0.052 | 0.239±0.074 | 0.063 |
| Cerebellum-White_L | 0.194±0.067 | 0.303±0.110 | 0.059 |
| Cerebellum-White_R | 0.211±0.076 | 0.290±0.095 | 0.133 |
| Colliculus Inferior_L | 0.115±0.043 | 0.208±0.075 | 0.020* |
| Colliculus Inferior_R | 0.121±0.048 | 0.246±0.102 | 0.018* |
| Thalamus_L | 0.088±0.032 | 0.152±0.050 | 0.020* |
| Thalamus_R | 0.101±0.031 | 0.165±0.052 | 0.021* |
| Pituitary | 0.404±0.153 | 0.623±0.268 | 0.105 |
| Cerebellum-blood | 0.314±0.114 | 0.420±0.165 | 0.213 |
| Central Canal-Periaqueductal Gray | 0.088±0.033 | 0.153±0.067 | 0.050 |
| Pons | 0.127±0.041 | 0.233±0.093 | 0.024* |
| Septum | 0.144±0.058 | 0.178±0.050 | 0.281 |
| Medulla | 0.168±0.054 | 0.278±0.070 | 0.010* |

Note: **P*<0.05 and ***P*<0.01 were regarded as statistically significant. BDL = bile duct ligation; L = left; R = right.
