# Supplementary material for: [18F]PBR146 and [18F]DPA-714 in vivo Imaging of Neuroinflammation in Chronic Hepatic Encephalopathy Rats
Source: Front Neurosci. 2021 Aug 16;15:678144. doi: 10.3389/fnins.2021.678144 (PMC8415356; doi:10.3389/fnins.2021.678144)
Supplement: Supplementary file 4 [file Table_3.docx]

## Supplementary table S3. Comparison of [^18^F]PBR146 uptake values in regional brain between Sham and BDL groups (%ID/g)

| **Brain Regions** | **Sham (n=6)** | **BDL (n=6)** | ***P*** |
| --- | --- | --- | --- |
| Accumbens_L | 0.113±0.032 | 0.171±0.086 | 0.152 |
| Accumbens_R | 0.085±0.020 | 0.201±0.113 | 0.032* |
| Amygdala_L | 0.191±0.032 | 0.231±0.072 | 0.248 |
| Amygdala_R | 0.171±0.045 | 0.242±0.081 | 0.090 |
| Striatum_L | 0.088±0.020 | 0.145±0.048 | 0.022* |
| Striatum_R | 0.108±0.011 | 0.175±0.085 | 0.083 |
| Auditory Cortex_L | 0.144±0.019 | 0.194±0.043 | 0.037* |
| Auditory Cortex_R | 0.141±0.020 | 0.212±0.080 | 0.083 |
| Cingulate Cortex_L | 0.103±0.016 | 0.192±0.097 | 0.074 |
| Cingulate Cortex_R | 0.095±0.025 | 0.282±0.031 | 0.169 |
| Entorhinal Cortex_L | 0.247±0.044 | 0.254±0.073 | 0.837 |
| Entorhinal Cortex_R | 0.212±0.044 | 0.241±0.077 | 0.431 |
| Frontal Association Cortex_L | 0.132±0.061 | 0.156±0.052 | 0.487 |
| Frontal Association Cortex_R | 0.107±0.046 | 0.295±0.035 | 0.216 |
| Insular Cortex_L | 0.151±0.032 | 0.180±0.068 | 0.375 |
| Insular Cortex_R | 0.134±0.035 | 0.185±0.059 | 0.103 |
| Medial Prefrontal Cortex_L | 0.079±0.021 | 0.189±0.145 | 0.096 |
| Medial Prefrontal Cortex_R | 0.096±0.027 | 0.264±0.032 | 0.230 |
| Motor Cortex_L | 0.112±0.018 | 0.158±0.041 | 0.033* |
| Motor Cortex_R | 0.103±0.032 | 0.235±0.023 | 0.192 |
| Orbitofrontal Cortex_L | 0.111±0.022 | 0.177±0.059 | 0.026* |
| Orbitofrontal Cortex_R | 0.119±0.032 | 0.275±0.026 | 0.181 |
| Para Cortex_L | 0.087±0.024 | 0.136±0.050 | 0.055 |
| Para Cortex_R | 0.090±0.034 | 0.155±0.059 | 0.041* |
| Retrosplenial Cortex_L | 0.135±0.031 | 0.211±0.065 | 0.037* |
| Retrosplenial Cortex_R | 0.152±0.036 | 0.197±0.065 | 0.166 |
| Somatosensory Cortex_L | 0.096±0.021 | 0.135±0.037 | 0.044* |
| Somatosensory Cortex_R | 0.107±0.021 | 0.152±0.038 | 0.033* |
| Visual Cortex_L | 0.128±0.013 | 0.180±0.051 | 0.054 |
| Visual Cortex_R | 0.117±0.020 | 0.196±0.044 | 0.003** |
| Hippocampus Antero Dorsal_L | 0.088±0.013 | 0.187±0.086 | 0.036* |
| Hippocampus Antero Dorsal_R | 0.109±0.023 | 0.215±0.090 | 0.019* |
| Hippocampus Posterior_L | 0.151±0.036 | 0.229±0.093 | 0.083 |
| Hippocampus Posterior_R | 0.127±0.045 | 0.264±0.141 | 0.045* |
| Hypothalamus_L | 0.139±0.042 | 0.225±0.092 | 0.065 |
| Hypothalamus_R | 0.122±0.033 | 0.209±0.065 | 0.015* |
| Olfactory_L | 0.196±0.035 | 0.238±0.092 | 0.317 |
| Olfactory_R | 0.162±0.054 | 0.238±0.095 | 0.115 |
| Colliculus Superior_L | 0.110±0.036 | 0.200±0.071 | 0.021* |
| Colliculus Superior_R | 0.107±0.034 | 0.166±0.091 | 0.173 |
| Midbrain_L | 0.093±0.007 | 0.201±0.139 | 0.116 |
| Midbrain_R | 0.094±0.021 | 0.176±0.085 | 0.066 |
| Ventral Tegmental Area_L | 0.125±0.033 | 0.238±0.129 | 0.085 |
| Ventral Tegmental Area_R | 0.095±0.027 | 0.211±0.104 | 0.025* |
| Cerebellum-Grey_L | 0.204±0.027 | 0.237±0.050 | 0.175 |
| Cerebellum-Grey_R | 0.177±0.015 | 0.230±0.065 | 0.084 |
| Cerebellum-White_L | 0.211±0.028 | 0.278±0.082 | 0.084 |
| Cerebellum-White_R | 0.201±0.014 | 0.300±0.087 | 0.038* |
| Colliculus Inferior_L | 0.085±0.026 | 0.203±0.070 | 0.007** |
| Colliculus Inferior_R | 0.136±0.056 | 0.208±0.072 | 0.081 |
| Thalamus_L | 0.089±0.023 | 0.172±0.090 | 0.075 |
| Thalamus_R | 0.089±0.016 | 0.165±0.092 | 0.098 |
| Pituitary | 0.416±0.117 | 0.522±0.227 | 0.340 |
| Cerebellum-blood | 0.303±0.062 | 0.477±0.170 | 0.055 |
| Central Canal-Periaqueductal Gray | 0.103±0.019 | 0.140±0.067 | 0.241 |
| Pons | 0.159±0.050 | 0.223±0.091 | 0.158 |
| Septum | 0.126±0.019 | 0.208±0.147 | 0.230 |
| Medulla | 0.180±0.020 | 0.263±0.071 | 0.034* |

Note: **P*<0.05 and ***P*<0.01 were regarded as statistically significant. BDL = bile duct ligation; L = left; R = right.
