# Supplementary material for: [18F]PBR146 and [18F]DPA-714 in vivo Imaging of Neuroinflammation in Chronic Hepatic Encephalopathy Rats
Source: Front Neurosci. 2021 Aug 16;15:678144. doi: 10.3389/fnins.2021.678144 (PMC8415356; doi:10.3389/fnins.2021.678144)
Supplement: Supplementary file 5 [file Table_4.docx]

## Supplementary table S4. The correlations between [^18^F]DPA-714 uptake values in global and regional brain and the results of behavior studies

| **Brain Regions** | **Time on the rotarod** | |  | **Beam walking cross time** | |  | **Corssovers of motor activity** | |
| --- | --- | --- | --- | --- | --- | --- | --- | --- |
|  | *P* | *r* |  | *P* | *r* |  | *P* | *r* |
| Global brain | 0.695 | -0.127 |  | 0.042* | 0.571 |  | 0.587 | -0.166 |
| Accumbens_L | 0.232 | -0.373 |  | 0.031* | 0.599 |  | 0.297 | -0.313 |
| Accumbens_R | 0.777 | 0.092 |  | 0.009** | 0.690 |  | 0.197 | -0.383 |
| Amygdala_L | 0.304 | -0.324 |  | 0.068 | 0.521 |  | 0.200 | -0.380 |
| Amygdala_R | 0.896 | -0.042 |  | 0.007** | 0.703 |  | 0.284 | -0.322 |
| Striatum_L | 0.678 | -0.134 |  | 0.001** | 0.797 |  | 0.422 | -0.244 |
| Striatum_R | 0.524 | -0.204 |  | 0.007** | 0.709 |  | 0.651 | -0.139 |
| Auditory Cortex_L | 0.948 | -0.021 |  | 0.022* | 0.626 |  | 0.364 | -0.275 |
| Auditory Cortex_R | 0.171 | -0.423 |  | 0.001** | 0.825 |  | 0.297 | -0.313 |
| Cingulate Cortex_L | 0.121 | -0.472 |  | 0.006** | 0.714 |  | 0.211 | -0.372 |
| Cingulate Cortex_R | 0.232 | -0.373 |  | 0.001** | 0.825 |  | 0.207 | -0.374 |
| Entorhinal Cortex_L | 0.481 | -0.225 |  | 0.007** | 0.709 |  | 0.279 | -0.325 |
| Entorhinal Cortex_R | 0.913 | -0.035 |  | 0.056 | 0.541 |  | 0.395 | -0.258 |
| Frontal Association Cortex_L | 0.584 | 0.176 |  | 0.116 | 0.458 |  | 0.711 | -0.114 |
| Frontal Association Cortex_R | 0.983 | 0.007 |  | 0.059 | 0.535 |  | 0.405 | -0.252 |
| Insular Cortex_L | 0.304 | -0.324 |  | 0.001** | 0.811 |  | 0.145 | -0.427 |
| Insular Cortex_R | 0.427 | -0.254 |  | 0.025* | 0.615 |  | 0.466 | -0.222 |
| Medial Prefrontal Cortex_L | 0.569 | -0.183 |  | 0.047* | 0.560 |  | 0.900 | -0.039 |
| Medial Prefrontal Cortex_R | 0.252 | -0.359 |  | 0.034* | 0.590 |  | 0.238 | -0.352 |
| Motor Cortex_L | 0.965 | -0.014 |  | 0.151 | 0.422 |  | 0.893 | -0.042 |
| Motor Cortex_R | 0.845 | 0.063 |  | 0.049* | 0.554 |  | 0.568 | -0.175 |
| Orbitofrontal Cortex_L | 0.760 | -0.099 |  | 0.006** | 0.717 |  | 0.427 | -0.241 |
| Orbitofrontal Cortex_R | 0.794 | -0.085 |  | 0.004** | 0.737 |  | 0.379 | -0.266 |
| Para Cortex_L | 0.983 | 0.007 |  | 0.154 | 0.419 |  | 0.871 | 0.050 |
| Para Cortex_R | 0.727 | 0.113 |  | 0.273 | 0.328 |  | 0.871 | 0.050 |
| Retrosplenial Cortex_L | 0.828 | 0.070 |  | 0.196 | 0.383 |  | 0.878 | 0.047 |
| Retrosplenial Cortex_R | 0.524 | -0.204 |  | 0.006** | 0.712 |  | 0.556 | -0.180 |
| Somatosensory Cortex_L | 0.599 | -0.169 |  | 0.101 | 0.474 |  | 0.808 | -0.075 |
| Somatosensory Cortex_R | 0.695 | -0.127 |  | 0.075 | 0.510 |  | 0.562 | -0.178 |
| Visual Cortex_L | 0.711 | 0.120 |  | 0.037* | 0.582 |  | 0.531 | -0.191 |
| Visual Cortex_R | 0.510 | -0.211 |  | 0.009** | 0.692 |  | 0.665 | -0.133 |
| Hippocampus Antero Dorsal_L | 0.121 | -0.472 |  | 0.052 | 0.549 |  | 0.422 | -0.244 |
| Hippocampus Antero Dorsal_R | 0.149 | -0.444 |  | 0.024* | 0.621 |  | 0.292 | -0.316 |
| Hippocampus Posterior_L | 0.454 | -0.239 |  | 0.052 | 0.549 |  | 0.725 | -0.108 |
| Hippocampus Posterior_R | 0.327 | -0.310 |  | 0.021* | 0.629 |  | 0.671 | -0.130 |
| Hypothalamus_L | 0.351 | -0.296 |  | 0.097 | 0.480 |  | 0.279 | -0.325 |
| Hypothalamus_R | 0.646 | -0.148 |  | 0.021* | 0.632 |  | 0.395 | -0.258 |
| Olfactory_L | 0.388 | -0.275 |  | 0.042* | 0.571 |  | 0.478 | -0.216 |
| Olfactory_R | 0.327 | -0.310 |  | 0.024* | 0.618 |  | 0.329 | -0.294 |
| Colliculus Superior_L | 0.948 | 0.021 |  | 0.071 | 0.516 |  | 0.864 | -0.053 |
| Colliculus Superior_R | 0.363 | -0.289 |  | 0.002** | 0.781 |  | 0.344 | -0.286 |
| Midbrain_L | 0.931 | -0.028 |  | 0.024* | 0.618 |  | 0.349 | -0.283 |
| Midbrain_R | 0.569 | -0.183 |  | 0.116 | 0.458 |  | 0.495 | -0.208 |
| Ventral Tegmental Area_L | 0.232 | -0.373 |  | 0.003** | 0.759 |  | 0.279 | -0.325 |
| Ventral Tegmental Area_R | 0.678 | -0.134 |  | 0.008** | 0.698 |  | 0.222 | -0.363 |
| Cerebellum-Grey_L | 0.845 | 0.063 |  | 0.048* | 0.557 |  | 0.794 | -0.080 |
| Cerebellum-Grey_R | 0.948 | 0.021 |  | 0.021* | 0.629 |  | 0.725 | -0.108 |
| Cerebellum-White_L | 0.760 | 0.099 |  | 0.128 | 0.444 |  | 0.950 | -0.019 |
| Cerebellum-White_R | 0.539 | -0.197 |  | 0.142 | 0.430 |  | 0.808 | 0.075 |
| Colliculus Inferior_L | 0.615 | 0.162 |  | 0.166 | 0.408 |  | 0.684 | -0.125 |
| Colliculus Inferior_R | 0.678 | -0.134 |  | 0.002** | 0.783 |  | 0.320 | -0.300 |
| Thalamus_L | 0.524 | -0.204 |  | 0.032* | 0.596 |  | 0.732 | -0.105 |
| Thalamus_R | 0.539 | -0.197 |  | 0.006** | 0.717 |  | 0.472 | -0.219 |
| Pituitary | 0.744 | -0.106 |  | 0.123 | 0.450 |  | 0.698 | -0.119 |
| Cerebellum-blood | 0.931 | 0.028 |  | 0.570 | 0.174 |  | 0.451 | 0.229 |
| Central Canal-Periaqueductal Gray | 0.495 | -0.218 |  | 0.002** | 0.767 |  | 0.507 | -0.203 |
| Pons | 0.440 | -0.246 |  | 0.039* | 0.577 |  | 0.531 | -0.191 |
| Septum | 0.711 | -0.120 |  | 0.095 | 0.483 |  | 0.921 | 0.031 |
| Medulla | 0.678 | -0.134 |  | 0.001** | 0.825 |  | 0.222 | -0.363 |

Note: **P*<0.05 and ***P*<0.01 were regarded as statistically significant. BDL = bile duct ligation; L = left; R = right.
