# Supplementary material for: [18F]PBR146 and [18F]DPA-714 in vivo Imaging of Neuroinflammation in Chronic Hepatic Encephalopathy Rats
Source: Front Neurosci. 2021 Aug 16;15:678144. doi: 10.3389/fnins.2021.678144 (PMC8415356; doi:10.3389/fnins.2021.678144)
Supplement: Supplementary file 6 [file Table_5.docx]

## Supplementary table S5. The correlations between [^18^F]PBR146 uptake values in global and regional brain and the results of behavior studies

| **Brain Regions** | **Time on the rotarod** | |  | **Beam walking cross time** | |  | **Corssovers of motor activity** | |
| --- | --- | --- | --- | --- | --- | --- | --- | --- |
|  | *P* | *r* |  | *P* | *r* |  | *P* | *r* |
| Global brain | 0.413 | -0.275 |  | 0.335 | 0.305 |  | 0.192 | -0.405 |
| Accumbens_L | 0.022* | -0.679 |  | 0.511 | 0.211 |  | 0.227 | -0.377 |
| Accumbens_R | 0.207 | -0.413 |  | 0.239 | 0.368 |  | 0.046* | -0.585 |
| Amygdala_L | 0.048 | -0.606 |  | 0.076 | 0.530 |  | 0.002** | -0.785 |
| Amygdala_R | 0.005** | -0.780 |  | 0.161 | 0.432 |  | 0.101 | -0.496 |
| Striatum_L | 0.106 | -0.514 |  | 0.736 | 0.109 |  | 0.125 | -0.468 |
| Striatum_R | 0.053 | -0.596 |  | 0.828 | 0.070 |  | 0.232 | -0.373 |
| Auditory Cortex_L | 0.024* | -0.670 |  | 0.111 | 0.484 |  | 0.200 | -0.398 |
| Auditory Cortex_R | 0.027* | -0.661 |  | 0.340 | 0.302 |  | 0.075 | -0.532 |
| Cingulate Cortex_L | 0.017* | -0.697 |  | 0.585 | 0.175 |  | 0.569 | -0.183 |
| Cingulate Cortex_R | 0.001** | -0.844 |  | 0.346 | 0.298 |  | 0.363 | -0.289 |
| Entorhinal Cortex_L | 0.022* | -0.679 |  | 0.070 | 0.540 |  | 0.002** | -0.789 |
| Entorhinal Cortex_R | 0.030* | -0.651 |  | 0.193 | 0.404 |  | 0.027* | -0.634 |
| Frontal Association Cortex_L | 0.446 | -0.257 |  | 0.511 | -0.211 |  | 0.794 | 0.085 |
| Frontal Association Cortex_R | 0.044* | -0.615 |  | 0.712 | 0.119 |  | 0.200 | -0.398 |
| Insular Cortex_L | 0.073 | -0.560 |  | 0.624 | 0.158 |  | 0.025* | -0.641 |
| Insular Cortex_R | 0.068 | -0.569 |  | 0.396 | 0.270 |  | 0.022* | -0.651 |
| Medial Prefrontal Cortex_L | 0.048* | -0.606 |  | 0.215 | 0.386 |  | 0.304 | -0.324 |
| Medial Prefrontal Cortex_R | 0.165 | -0.450 |  | 0.897 | 0.042 |  | 0.293 | -0.331 |
| Motor Cortex_L | 0.019* | -0.688 |  | 0.720 | 0.116 |  | 0.407 | -0.264 |
| Motor Cortex_R | 0.003** | -0.798 |  | 0.346 | 0.298 |  | 0.277 | -0.342 |
| Orbitofrontal Cortex_L | 0.004** | -0.789 |  | 0.526 | 0.204 |  | 0.615 | -0.162 |
| Orbitofrontal Cortex_R | 0.009** | -0.743 |  | 0.274 | 0.344 |  | 0.407 | -0.264 |
| Para Cortex_L | 0.113 | -0.505 |  | 0.812 | -0.077 |  | 0.488 | -0.222 |
| Para Cortex_R | 0.068 | -0.569 |  | 0.570 | 0.182 |  | 0.160 | -0.433 |
| Retrosplenial Cortex_L | 0.044* | -0.615 |  | 0.854 | 0.060 |  | 0.447 | -0.243 |
| Retrosplenial Cortex_R | 0.207 | -0.413 |  | 0.879 | -0.049 |  | 0.149 | -0.444 |
| Somatosensory Cortex_L | 0.085 | -0.541 |  | 0.812 | 0.077 |  | 0.183 | -0.412 |
| Somatosensory Cortex_R | 0.019* | -0.688 |  | 0.585 | 0.175 |  | 0.257 | -0.356 |
| Visual Cortex_L | 0.207 | -0.413 |  | 0.965 | -0.014 |  | 0.488 | -0.222 |
| Visual Cortex_R | 0.156 | -0.459 |  | 0.845 | 0.063 |  | 0.592 | -0.173 |
| Hippocampus Antero Dorsal_L | 0.006** | -0.761 |  | 0.371 | 0.284 |  | 0.128 | -0.465 |
| Hippocampus Antero Dorsal_R | 0.085 | -0.541 |  | 0.570 | 0.182 |  | 0.454 | -0.239 |
| Hippocampus Posterior_L | 0.044* | -0.615 |  | 0.229 | 0.375 |  | 0.080 | -0.525 |
| Hippocampus Posterior_R | 0.068 | -0.569 |  | 0.253 | 0.358 |  | 0.060 | --0.556 |
| Hypothalamus_L | 0.033* | -0.642 |  | 0.177 | 0.418 |  | 0.019* | -0.662 |
| Hypothalamus_R | 0.003** | -0.798 |  | 0.114 | 0.481 |  | 0.066 | -0.546 |
| Olfactory_L | 0.063 | -0.578 |  | 0.448 | 0.242 |  | 0.026* | -0.637 |
| Olfactory_R | 0.027* | -0.661 |  | 0.074 | 0.533 |  | 0.011* | -0.704 |
| Colliculus Superior_L | 0.106 | -0.514 |  | 0.570 | 0.182 |  | 0.272 | -0.345 |
| Colliculus Superior_R | 0.121 | -0.495 |  | 0.897 | 0.042 |  | 0.101 | -0.496 |
| Midbrain_L | 0.068 | -0.569 |  | 0.198 | 0.400 |  | 0.048* | -0.581 |
| Midbrain_R | 0.058 | -0.587 |  | 0.812 | -0.077 |  | 0.503 | -0.215 |
| Ventral Tegmental Area_L | 0.002** | -0.817 |  | 0.248 | 0.361 |  | 0.227 | -0.377 |
| Ventral Tegmental Area_R | 0.092 | -0.532 |  | 0.728 | 0.112 |  | 0.546 | -0.194 |
| Cerebellum-Grey_L | 0.006** | -0.761 |  | 0.448 | 0.242 |  | 0.058 | -0.560 |
| Cerebellum-Grey_R | 0.006** | -0.761 |  | 0.519 | 0.207 |  | 0.066 | -0.546 |
| Cerebellum-White_L | 0.015 | -0.706 |  | 0.664 | 0.140 |  | 0.051 | -0.574 |
| Cerebellum-White_R | 0.037* | -0.633 |  | 0.957 | -0.018 |  | 0.433 | -0.250 |
| Colliculus Inferior_L | 0.037* | -0.633 |  | 0.448 | 0.242 |  | 0.247 | -0.363 |
| Colliculus Inferior_R | 0.005** | -0.780 |  | 0.664 | 0.140 |  | 0.232 | -0.373 |
| Thalamus_L | 0.012* | -0.725 |  | 0.720 | 0.116 |  | 0.678 | -0.134 |
| Thalamus_R | 0.030* | -0.651 |  | 0.497 | 0.218 |  | 0.048* | -0.581 |
| Pituitary | 0.307 | -0.339 |  | 0.483 | 0.225 |  | 0.055 | -0.567 |
| Cerebellum-blood | 0.003** | -0.798 |  | 0.648 | 0.147 |  | 0.623 | -0.158 |
| Central Canal-Periaqueductal Gray | 0.099 | -0.523 |  | 0.632 | 0.154 |  | 0.205 | -0.394 |
| Pons | 0.147 | -0.468 |  | 0.317 | 0.316 |  | 0.056 | -0.563 |
| Septum | 0.207 | -0.413 |  | 0.215 | 0.386 |  | 0.092 | -0.507 |
| Medulla | 0.002** | -0.817 |  | 0.435 | 0.249 |  | 0.272 | -0.345 |

Note: **P*<0.05 and ***P*<0.01 were regarded as statistically significant. BDL = bile duct ligation; L = left; R = right.
